# Comparing pharmacophore models derived from crystal structures and from molecular dynamics simulations

**DOI:** 10.1007/s00706-016-1674-1

**Published:** 2016-02-22

**Authors:** Marcus Wieder, Ugo Perricone, Thomas Seidel, Stefan Boresch, Thierry Langer

**Affiliations:** Department of Pharmaceutical Chemistry, Faculty of Life Sciences, University of Vienna, Vienna, Austria; Department of Computational Biological Chemistry, Faculty of Chemistry, University of Vienna, Vienna, Austria; Dipartimento di Scienze e Tecnologie Biologiche Chimiche e Farmaceutiche “STEBICEF”, Università di Palermo, Palermo, Italy

**Keywords:** Pharmacophore modelling, Molecular dynamics, Molecular modelling, Computational chemistry

## Abstract

**Abstract:**

Pharmacophore modeling is a widely used technique in computer-aided drug discovery. Structure-based pharmacophore models of a ligand in complex with a protein have proven to be useful for supporting in silico hit discovery, hit to lead expansion, and lead optimization. As a structure-based approach it depends on the correct interpretation of ligand–protein interactions. There are legitimate concerns about the fidelity of the bound ligand and about non-physiological contacts with parts of the crystal and the solvent effects that influence the protein structure. A possible way to refine the structure of a protein–ligand system is to use the final structure of a given MD simulation. In this study we compare pharmacophore models built using the initial protein–ligand structure obtained from the protein data bank (PDB) with pharmacophore models built with the final structure of a molecular dynamics simulation. We show that the pharmacophore models differ in feature number and feature type and that the pharmacophore models built from the last structure of a MD simulation shows in some cases better ability to distinguish between active and decoy ligand structures.

**Graphical abstract:**

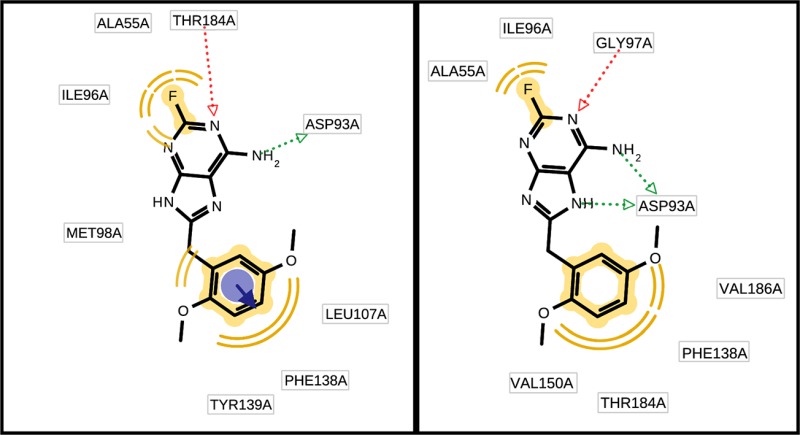

## Introduction

The aim of this study is to compare pharmacophore models obtained from the crystal structure of a ligand–protein complex with the pharmacophore models derived from the last frame of a molecular dynamics (MD) simulation. In the following, the pharmacophore model obtained from the crystal structure of a ligand–protein complex will be called initial pharmacophore model, the pharmacophore models derived from the last frame of a MD simulation will be called MD-refined pharmacophore model. Considering the final structure of a given MD simulation is the most basic and straightforward MD-based structure refinement protocol; although simple it can resolve some of the problems connected to protein–ligand structures obtained from X-ray crystallography [1–3]. We believe that considering the initial pharmacophore model together with the MD-refined models can give valuable additional information for constructing pharmacophore models.

We investigated two key questions: (1) are the pharmacophore model obtained from the crystal structure different from the pharmacophore model obtained from the final structure of the MD simulation? (2) is there a difference in the ability of the initial pharmacophore model and the MD-refined pharmacophore model to distinguish between active and decoy compounds?

The first question was answered by visual inspection of the obtained pharmacophore models. To answer the second question we screened active/decoy databases of the investigated protein–ligand complexes to calculate receiver operating characteristic (ROC) curves and enrichment factors [4, 5].

### Pharmacophore modelling

Structure-based pharmacophore models of a ligand in complex with a protein have proven to be useful for supporting in silico hit discovery, hit to lead expansion, and lead optimization [6]. Pharmacophore models are defined as the ensemble of steric and electronic features that are necessary to ensure the optimal supramolecular interactions with a specific biological target structure and to trigger or block its biological response [7]. These features include H-bond acceptors, H-bond donors, positive and negative ionizable groups as well as lipophilic regions and aromatic rings. The protocol for the generation of structure-based pharmacophore models involves the analysis of the complementary chemical features of the 3D structure of the active site and their spatial relationship to assemble the pharmacophore model. The aim of pharmacophore modelling is to gain insights into ligand–protein interactions, to retrieve the essential pharmacophore features necessary for optimal interaction, and to identify novel compounds that satisfy steric and electrostatic requirements with a high probability of biological activity [8, 9]. Structure-based pharmacophore models can be generated using a variety of software packages including Schrodinger [10], FLAP [11], GBPM [12], HS-Pharm [13], and LigandScout [14]. The starting point of structure-based pharmacophore models are usually the coordinates of a reference protein–ligand complex obtained from the protein data bank (PDB) [15]. Around 90 % of these coordinate files are generated using X-ray crystallography. For structure-based modelling it is mandatory that these structures are correct with respect to bond length and angles. There are legitimate concerns about the fidelity of the bound ligand and about non-physiological contacts with parts of the crystal and the solvent effects that influence the protein structure [2, 16–19]. This fact can lead to pharmacophore models that are not representative for the protein–ligand interaction pattern in vivo. A possible way to refine the structure of a protein–ligand system is to use the final structure of a given MD simulation [1].

### Molecular dynamics simulations

MD simulation is a computational technique to solve Newton’s equation of motions for a given system of atoms. This technique is widely used to obtain information about the coordinates of a protein–ligand system as a function of time. MD simulations can provide detailed information concerning the dynamics of atoms and molecules and give insights into dynamic properties, solvent effects and free energy of protein/ligand binding of a model system [20–22]. MD has been widely applied in the field of drug discovery [23, 24].

In this study MD simulations are used to obtain the final protein–ligand structure after 20 ns of simulation time. Molecular dynamics simulations with reasonable initial velocity follow the path of steepest descent on the potential energy surface to a local minimum [25]. Subsequently the protein–ligand system is trapped if the confining barriers are significant at the simulation temperature and therefore the region on conformational space surrounding this minimum becomes the most populated region [26].

MD-based approaches to refine protein structures and regard receptor flexibility are well established for various modelling methods, especially for molecular docking (see e.g. [27, 28]).

### Systems used for MD simulations

For analysis, six different protein–ligand systems with PDB code 1J4H, 3BQD, 2HZI, 3L3 M, 1UYG, and 3EL8 were chosen from the DUD-E database. This database provides known actives and decoys that are calculated using similar 1-D physico-chemical properties as the actives (e.g. molecular weight, calculated LogP) but dissimilar 2-D topology (based on ECFP4 fingerprints) [29]. The choice of complexes was somewhat arbitrary, though guided by the following considerations: system size (solvated protein–ligand complex less than 70,000 atoms), only a single ligand, no metal ions involved in the binding.

**Fig. 1:**
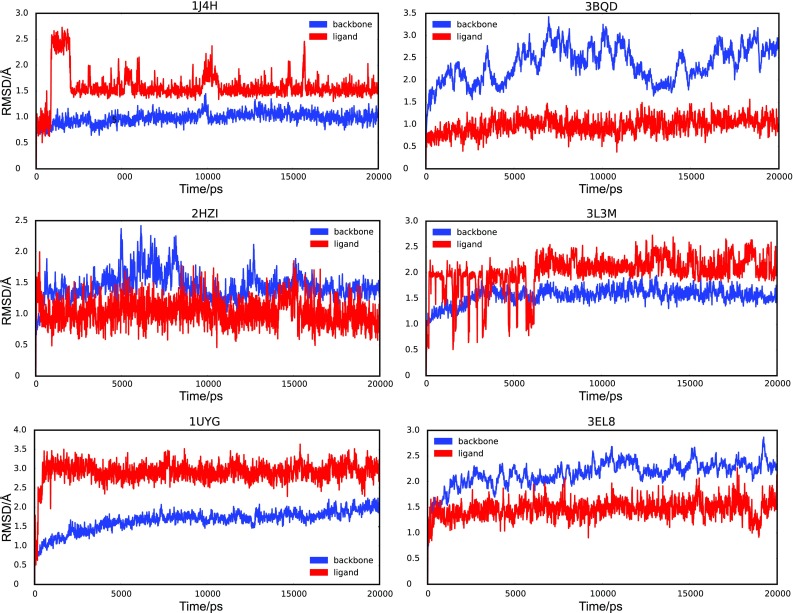
The root mean square deviation (RMSD) of the protein (in *red*) and the ligand (in *blue*) is provided as a function of time for the six analyzed protein–ligand complexes. The RMSD is calculated as described in the method section. For all systems the ligand and the protein experiences a rapid RMSD deviation from the original structure of at least 0.5 Å. The different RMSD ranges on the y-axis should be noted

Subsequently details on the different protein–ligand systems are provided (the structure of the ligand can be seen in Fig. 1):

- 1J4H (FKBP12 or FK506 binding protein) is a mostly hydrophilic monomeric prolyl isomerase with a molecular weight of 12 kDa which binds the immunosuppressant molecule tacrolimus (FK506) and is present in homo sapiens [30]. Previous studies have demonstrated that FKBP12 does not undergo significant conformation variations [31], small spatial rearrangements have been seen in a remote zone of the protein by Choi et al. [32]. The activity of the ligand is not known.
- 2HZI is the resolved X-ray structure of the human Abl kinase domain, a monomeric non-receptor tyrosine-protein kinase with a molecular weight of 32 kDa, in complex with PD180970. The BCR-Abl protein plays a role in many key processes linked to cell growth and survival and is located in the cytoplasm, nucleus and mitochondria [33]. The Abl kinase is a rather flexible protein even though in contact with high-affinity ligands (like PD180970) the stability is increased. The ligand has an IC50 of 70 nM [34].
- 3EL8 is the crystal structure of the protooncogen c-Src in complex with pyrazolopyrimidine from *Gallus gallus. C*-*Src is* a cytoplasmic non-receptor tyrosine kinase. 3EL8 in complex with its inhibitor show the energetically unfavored, inactive but stable (Asp-Phe-Gly)-out (DFG-out) conformation [35, 36]. The ligand has an IC50 of 25 nm [35].
- 1UYG is the crystal structure of the human HSP90-alpha N-terminal ATPase domain consisting of 236 amino acids with a molecular weight of 27 kDa. HSP90-alpha is a member of a highly abundant family of human chaperones responsible for the maturation and activity of a variety of key proteins involved in cell growth and proliferation [37, 38]. In previous studies it was shown that HSP90 is a highly dynamic and flexible molecule that can adopt a wide variety of structurally distinct states. These changes are ATP-dependent and influence the whole protein, the N-terminal domain alone appears to be stable [39]. The ligand has an IC50 of 53 μM [38].
- 3BQD is the crystal structure of the nuclear receptor ligand-binding domain of the human glucocorticoid receptor with a length of 255 amino-acids and a S602F mutation. It is a globular domain with 11 alpha-helices, 4 beta-strands and a molecular weight of 31 kDa located in the cytoplasm and nucleus. The domain is co-crystallized with deacylcortivazol. The binding of this ligand to the glucocorticoid receptor expands the binding pocket yet leaving the structure of the coactivator binding site intact. This shows that nuclear receptors have a great degree of conformational capacity [40]. There is no binding data available for the ligand.
- The crystal structure 3L3 M is a subunit obtained from the poly(ADP-ribose) polymerase (PARP)-1 in complex with A927929. It involves the PARP alpha helical and PARP catalytical motif with a molecular weight of 39 kDa. PARPs are a family of nuclear enzymes involved in detection and repair of DNA damage [41]. The D-loop and four alpha-helices exhibit higher structural flexibility as has been shown in MD simulations [42]. There is no binding data available for the ligand.

For the rest of the article the protein systems will be referred to by their PDB code. The quality of all PDB structures was manually checked and models were corrected if necessary.

### Virtual screening with pharmacophore models

The virtual screening process uses the pharmacophore model as a query for classification of compounds into decoy and active compounds, assigns score values, and constructs a sorted list of these compounds using the score as key. The elements in the list are sorted from highest to lowest scores, higher score indicate that a molecule is assessed by the screening model as a potential active compound. A ROC curve is used to visualise this list, the rate of active compounds on the* X* axis and the rate of decoy compounds on the* Y* axis. A ROC curve that follows the dotted diagonal line represents an insignificant (random) classification model that cannot distinguish between decoy and active ligands. A ROC curve that is plotted above the diagonal represents a pharmacophore model that can detect actives [9].

The enrichment factor describes—in the context of pharmacophore models—the number of active compounds found by using a specific pharmacophore model as opposed to the number hypothetically found if compounds were screened randomly [43–45]. The enrichment criterion is evaluated by a numerical factor as defined in Eq. (1).

1 $$ {\text{EFsubset }} = \, \left( {tp_{\text{hitlist}} / \, tp_{\text{hitlist}} + \, fp_{\text{hitlist}} } \right) / \left( {N_{\text{A}} /N_{\text{A}} + \, N_{\text{D}} } \right) $$

where *tp*_hitlist_ is the number of true positive in the hitlist and *fp*_hitlist_ corresponds to the number of false positive in the hitlist. *N*_A_ and *N*_D_ are the number of active and decoy compounds in the testset. Enrichment factors can range from 1—which means that molecules are sorted randomly—to >100, which means that only a small percentage of the order list needs to be screened in vitro to find a large number of active molecules [5].

## Results and discussion

### Quality control of protein–ligand structures

For one protein (3EL8) it was necessary to add missing residues. Using the software Modeller 13 residues from residue number 411–423 were inserted [46, 47]. The amino acid sequence was obtained from the DNA sequence of the protein from the NCBI database [48]. The protonation state and side chain orientation was set in accord with propka [49, 50] and the quality control check provided by the joint center for structural genomics [51].

### Rmsd

For all protein–ligand systems, the root mean square deviation (RMSD) for the protein and the ligand was independently calculated and is shown in Fig. 1. The ligand and the protein RMSD values were calculated with the aligned C-alpha atoms of the target and reference structure.

The RMSD of the protein and ligand was analysed to detect large-scale movements of the protein or the ligand. In addition, we used the deviation of the ligand to determine if the ligand reaches a stable binding state. The RMSD plots of the different ligands show very similar behaviour. The RMSD usually changes in the beginning to an average value from which the ligand deviates only marginally. This transition happens fast, e.g. 2HZI reaches the average value of 1.03 Å in less than 0.1 ns and has a standard deviation of 0.2 Å from the mean. The ligand of 1J4H is the only exception—it takes nearly 2.5 ns to reach the stable plateau around the average value of 1.58.

For the protein the behaviour of the RMSD was in the range of normal conduct during a MD simulation.

### Comparing pharmacophore models

**Fig. 2:**
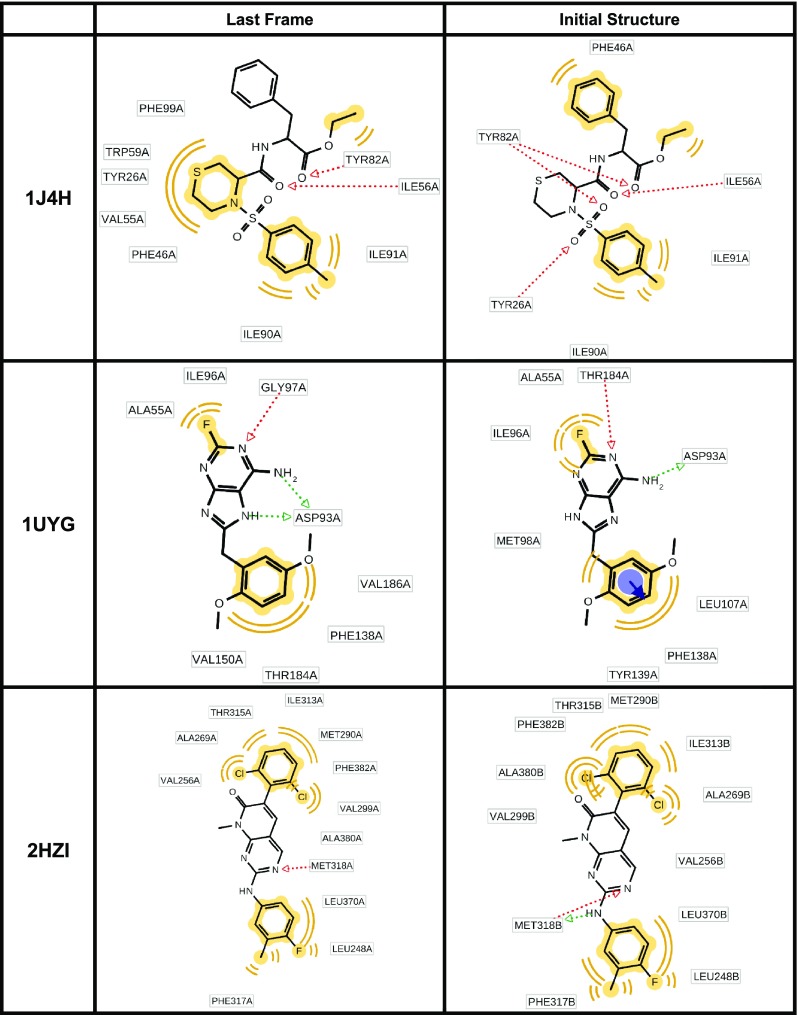

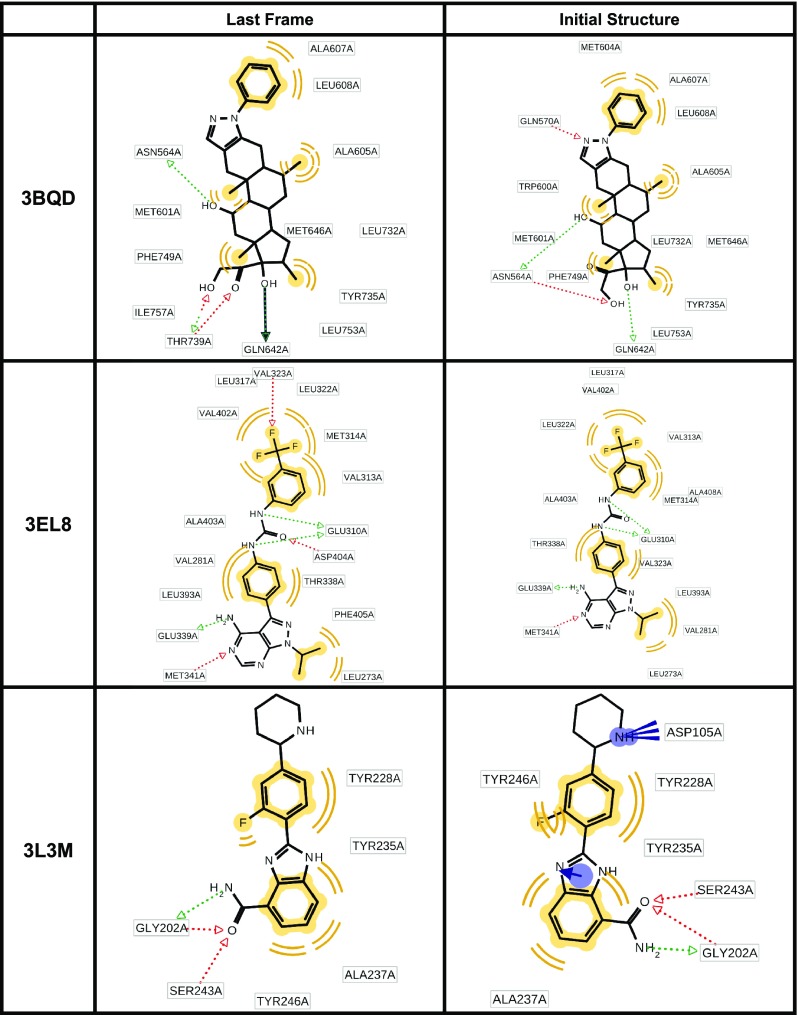
Comparing the initial pharmacophore model and the MD-refined pharmacophore model. The features in *yellow* indicate hydrophobic features, the vector features in *red* indicate hydrogen bond acceptors, the vector features in *green* indicate hydrogen bond donors, the feature spheres in *blue* with associated vectors indicate aromatic features and the features in *blue* with *multiple lines* associated indicate salt bridges

In Fig. 2 we report the 2D view of the ligand together with the assigned pharmacophore features. The pharmacophore model obtained from the PDB file and the MD-refined pharmacophore model are shown for every protein–ligand system.

For all analyzed systems the initial pharmacophore model and the MD-refined pharmacophore model differ. Of the six analyzed systems the amount of pharmacophore features for the initial model decreased in three cases, in one case the amount of features (but not the kind of features) stayed the same and in two cases the amount of features increased compared to the pharmacophore model obtained with the MD-refined pharmacophore model. Looking at specific feature types it is interesting to note that hydrophobic features do not change (with the notable exception of 1J4H) in amount nor in involved ligand atoms. In contrast none of the aromatic features are present in the MD-refined pharmacophore model. Most of the variability in the pharmacophore features was found to be due to hydrogen bond acceptors and donors.

### Virtual screening results

It should be mentioned that the following paragraph deals with the default pharmacophore models without any manual refinement. For drug discovery the default pharmacophore model would be submitted to various refinement steps to yield better results—since these steps depend heavily on the knowledge of the researcher performing the modeling it could bias a comparison of the screening results and was therefore omitted.

An additional issue that should be kept in mind is that the DUD-E database uses known actives but calculated decoys—that means that there is a chance that some decoys might still bind to the protein. Also, as a result of the decoy calculating process, decoys are often similar to the active molecules.

**Fig. 3:**
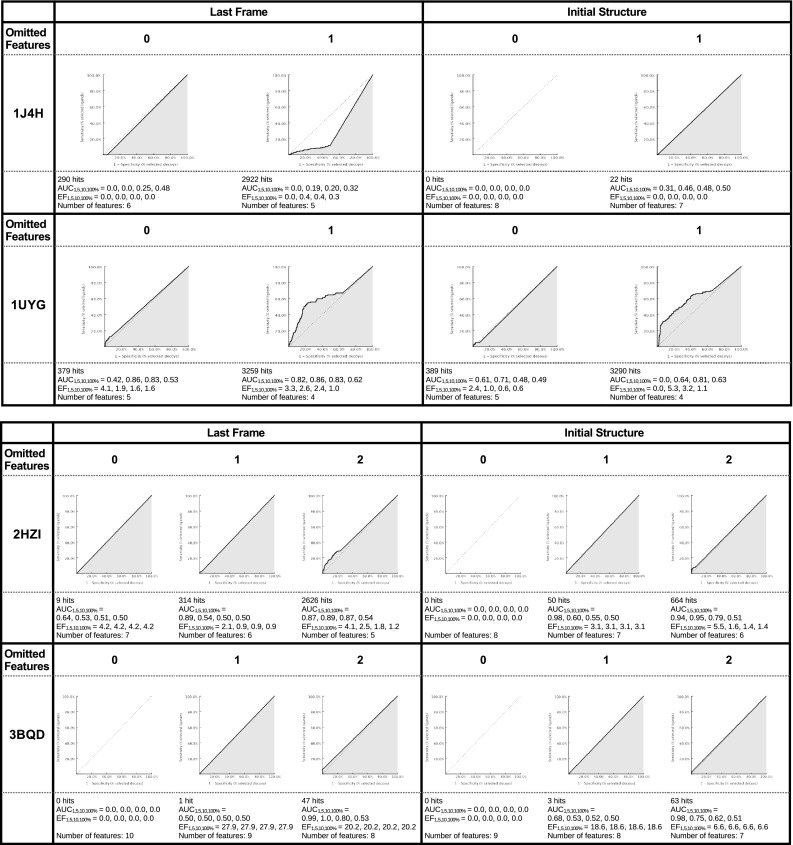

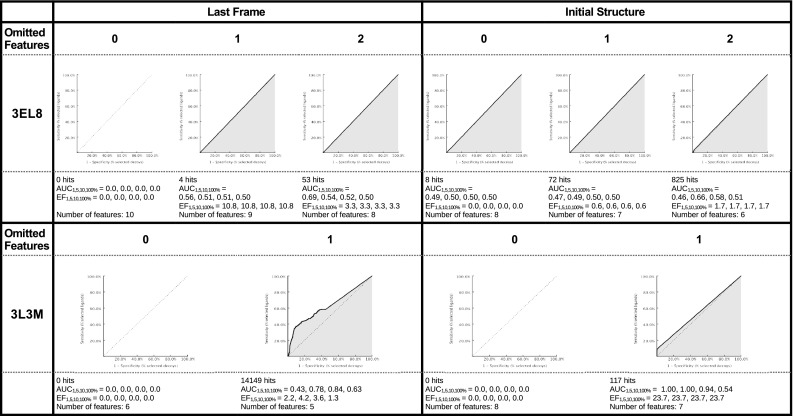
The receiver operating characteristic (ROC) curve for the different protein–ligand systems is shown. The true positive rate is seen on the Y axis and the false positive rate on the X axis. The number next to the PDB code indicates the number of omitted features: *0* means that no features were omitted, *1* or *2* means that either one or two features were omitted during the screening. In the plots the number of total hits, the area under the curve (AUC) and the enrichment factor (EF) is shown at 1, 5, 10 and 100 %

In Fig. 3 the ROC curves, the enrichment factor (EF), the area under the curve (AUC), and the number of features for the different pharmacophore models are shown. The MD-refined and the initial pharmacophore model for all protein–ligand systems (with the notable exception of 1J4H) are able to retrieve the original ligand (which is not part of the screening library).

- For 1J4H the initial pharmacophore model and the MD-refined pharmacophore model cannot distinguish between actives and decoys.
- For 1UYG the MD-refined pharmacophore model can distinguish between active and decoy compounds. With one omitted feature the overall ability to separate actives and decoys is better than with zero omitted features, but the enrichment factor for the first percent is lower. The initial pharmacophore model with zero omitted features can distinguish between active and decoy compounds for the first percent of the results, but above the 5 % mark it favors decoys over actives. The model with one omitted features has among the top ranking results only false positive compounds but after the 1 % mark it favors actives over decoys.
- The MD-refined pharmacophore model for 2HZI favors active over inactive compounds for zero, one, and two omitted features. This is not always visible in the ROC curve but looking at the enrichment factor it becomes clear that even the pharmacophore model with zero omitted features favors actives. The model with one omitted features favors actives only in the highest ranking results, the model with two omitted features favors actives for all results. The initial pharmacophore model with one and two omitted features favors actives.
- For 3BQD the MD-refined pharmacophore model with one and two omitted features has high enrichment factors (27.9 and 20.2 for 100 %) as well as the initial pharmacophore model with one or two omitted features of 18.6 and 6.6 for 100 %.
- For 3EL8 the MD-refined pharmacophore model with one and two omitted features has high enrichment factors (10.8 and 3.3 for 100 %) whereas the initial pharmacophore model with zero or one omitted features has no preference for actives, with two omitted features the model has a slight overall preference for active compounds (ER: 1.7 for 100 %).
- The MD-refined pharmacophore model for 3L3 M with zero omitted features has an overall preference for actives, but this effect is only marginal. The same model with one omitted features has a significant early enrichment but the sensitivity decreases after the 5 % mark. The initial pharmacophore model with zero omitted features is not able to return any results, with one omitted feature the model has a good early enrichment (23.7 %) with constant sensitivity.

The screening results obtained from the MD-refined pharmacophore model and from the initial pharmacophore model are different. With the exception of 1J4H, for which both pharmacophore models performed badly, either the refined pharmacophore model or the initial pharmacophore model were able to favor active compounds over inactive ones—in some cases, e.g. 3BQD both were able to distinguish between the groups. Depending on the preferred result (early enrichment vs overall enrichment factor) the interpretation of the overall performance of the two approaches can vary. Simply looking at early enrichment (considering only the enrichment factor at 1 % of the total compounds) the pharmacophore model obtained with the MD-refined pharmacophore model performs better for 1UYG as well as on average for 2HZI, 3BQD, and 3EL8. The initial pharmacophore model performs better for the screening on the compounds for 3L3 M.

Considering the enrichment factor at 100 % of analyzed compounds the MD-refined pharmacophore model performs better for 1J4H (even though still badly), as well as on average for 1UYG, 2HZI, and 3BQD. In the analysed cases the overall enrichment factor mirrors the results obtained from the early enrichment results.

It can be argued that the increased performance of the MD refined pharmacophore model of 1UYG is a result of the structural movement of the ligand as seen in Fig. 1—but since the MD refined pharmacophore model for 3BQD (which has very low RMSD values) performs better in virtual screening as well this line of reasoning was not followed. There is no obvious connection between high ligand RMSD (as seen for 1UYG, 3L3 M), medium RMSD (as seen for 3EL8, 2HZI, 1J4H), low RMSD (as seen for 3BQD) and performance in virtual screening. The same argument can be applied looking at protein RMSD values—there is no trend between the RMSD values for the protein and the performance of the pharmacophore model.

## Conclusion

The findings reported in this study suggest that even very simple structure refinement approaches—like the reported one—can lead to pharmacophore models that perform better in virtual screening. The refinement of pharmacophore models using molecular dynamics simulations is expedient in more than 50 % of the cases. For some of the protein–ligand complexes MD refinement did not yield better results—in these cases additional operations are necessary to improve the pharmacophore models.

The results shown indicate that additional interaction information can be unveiled from an analysis of the dynamics of protein and ligand. Using these information can lead to better pharmacophore models that can target specific binding sites or interact with transitional conformations.

It was not possible to find correlations between the performance increase of MD refined pharmacophore models and protein/ligand structure, RMSD values or number of pharmacophore features. Additional work is needed to find guidelines for MD structure optimization related to pharmacophore modeling.

## Methods

### Charmm

We used CHARMM-GUI to set up the simulations and the CHARMM software package to run them [52, 53]. The CGenFF and paramchem was used to obtain parameter and topology files for the small molecules [54, 55]. For all the CHARMM/OpenMM version was used to run molecular dynamics simulations for six protein–ligand complexes [56]. The systems were solvated in rectangular water boxes with TIP3P water molecules. Electrostatic interactions were computed by the particle-mesh-Ewald method. From the starting structures we carried constant pressure, constant temperature MD simulations (Berendsen thermostat and barostat). The length of each simulations was 20 ns; the time step was 2 fs and SHAKE was used to keep all bonds involving hydrogen atoms fixed. Before each simulation we equilibrated the protein–ligand–water system for 25 ps with a time step length of 1 fs.

### RMSD calculation

The RMSD was analysed using the python package MDAnalysis [57]. The RMSDs were calculated as follows: all coordinates saved during the MD were fitted against the starting structure based on the coordinates of the C_α_-atoms of the protein. Using the starting structure as reference, for these reoriented coordinates the RMSD of the C_α_-atoms was calculated for the protein and the RMSD of the heavy atoms of the ligand.

### LigandScout

For generating structure-based pharmacophore models and screening libraries LigandScout 4.09.1 was used. The screening libraries for the systems were generated using the decoys and actives from the DUD-E database [29].

All molecules were prepared as libraries for the screening using the command line tool idbgen provided by LigandScout (see Table 1 for the number of actives and decoys in the screening libraries). Conformers were generated using the icon best option in idbgen, this option produces a maximum number of 200 conformations for each molecule processed. Screening was performed using the command line tool iscreen provided by LigandScout [14].

**Table 1:** Number of actives and decoys obtained from the DUD-E database and used to construct the screening libraries

PDB CODE	Nr. of actives	Nr. of decoys
1J4H	273	5832
1UYG	124	4936
2HZI	293	10,879
3BQD	563	15,161
3EL8	823	34,873
3L3M	742	30,400

### PDB quality control

The quality and correctness of the PDB structures were audited using the Quality Control server [51]. Modeller 9.15 was used if residues were missing [47]. Subsequently all structures were analysed with PropKa 3.1 to check the protonation state of the protein and the ligand [49].
